# Tumor Microenvironment-Responsive Nanoplatform of Cu-Doped ZIF‑8 Dual-Loaded with ICG and DOX for Photothermal-Enhanced Chemodynamic Therapy/Chemotherapy

**DOI:** 10.1021/acsomega.5c11491

**Published:** 2026-02-11

**Authors:** Tao Yang, Tao Wang, Tao Shen, Mingrong Dong, Jinkun Liu, Ming Ni, Yan Zhu

**Affiliations:** † Faculty of Materials Science and Technology, 47910 Kunming University of Science and Technology, Yunnan 650093, China; ‡ Faculty of Information Engineering and Automation, Kunming University of Science and Technology, Kunming 650500, China; § Department of Orthopaedics, Shanghai Key Laboratory for Prevention and Treatment of Bone and Joint Diseases, Shanghai Institute of Traumatology and Orthopaedics, Ruijin Hospital, Shanghai Jiao Tong University School of Medicine, Shanghai 200025, China; ∥ Siluzhisheng (Beijing) Intelligent Technology Co., Ltd., Miyun District, Beijing 101500, China

## Abstract

Targeting tumor-specific characteristics of acidic microenvironment, elevated H_2_O_2_ levels, and thermosensitivity, this study proposed and developed a copper-doped ZIF-8 nanoplatform coloaded with indocyanine green (ICG) and doxorubicin hydrochloride (DOX), designated as Cu-ZIF-8@ICG&DOX (CZID), to establish a multimodal therapeutic system integrating chemodynamic therapy, photothermal therapy, and chemotherapy. Results showed that the 25% copper doping level optimized the structure, achieving 7.46% actual doping content while limiting the average nanoparticle size to 99.6 nm. Dual loading of ICG and DOX induced morphological transition to spherical core–shell architectures, as confirmed by zeta potential reversal, with maximum drug loading capacities of 23.01 μg/mg for ICG and 122.43 μg/mg for DOX. The ZIF-8 framework exhibited pH-responsive degradation under acidic conditions. Released Cu^2+^ ions mediated continuous hydroxyl radical generation through glutathione-depletion redox cycling, confirming chemodynamic efficacy. Under 808 nm laser irradiation, ICG enabled enhanced photothermal conversion, showing concentration- and power-dependent temperature elevation. Cellular assays revealed efficient CZID internalization by MCF-7 cells, generating significantly higher intracellular reactive oxygen species levels under irradiation and more intense dead cell staining compared to CZI and CZD groups. Such an enhancement was attributed to photothermally accelerated ZIF-8 degradation, promoting Cu^2+^ ions and DOX release, thereby strengthening chemodynamic–chemotherapy synergy. At the working concentration of 50 μg/mL, CZID induced 95.36% apoptosis in MCF-7 cells while maintaining over 70% viability in MCF-10A normal cells, validating its precise antitumor potential.

## Graphical abstract

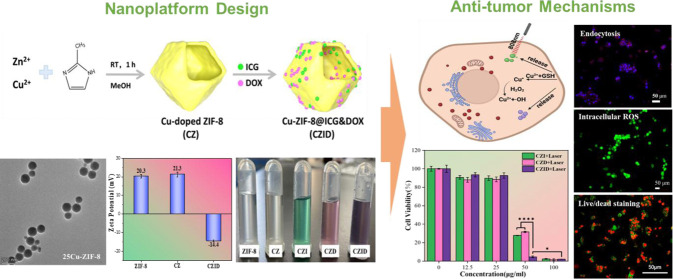

## 1 Introduction

Oncological disorders, most notably cancers, constitute a major disease category that poses a significant threat to human public health. It is reported that cancers claimed 9.7 million global lives in 2022, and this trend is becoming increasingly severe as the number of new cancer cases is projected to reach 35 million by 2050.^1^ Current cancer treatments exemplified by surgical removal and chemotherapy merely demonstrate a cure rate of less than 50%, and their successful outcomes mainly rely on early screening and diagnosis.^2^ Moreover, surgical removal and chemotherapy often cause residual problems like the irreversible damage to peri-tumoral normal tissues, functional organ deficits, and long-term systemic complications.^3,4^ Facing the constraints of traditional cancer treatments, more advanced therapies are expected to be developed.

Tumor physiology studies confirm that pathological hyperproliferation induces sustained hypoxia, compelling tumor cells to adopt anaerobic glycolysis; consequent lactate accumulation and respiratory CO_2_ establish a tumor microenvironment (TME) with marked weak acidity, elevated hydrogen peroxide (H_2_O_2_), and thermosensitivity.^5,6^ Based on this pathological signature, chemodynamic therapy (CDT) exploits transition metals (Fe, Co, and Mn) to catalyze the Fenton/Fenton-like reaction of GSH/H_2_O_2_, generating cytotoxic hydroxyl radicals (^•^OH) and oxidized glutathione (GSSG). This process elevates intracellular reactive oxygen species (ROS), inducing lethal DNA damage and apoptosis.^7^ Xie et al.^8^ synthesized 264 nm hollow CoFe_2_O_4_ nanoparticles loaded with Pt (CF@Pt NPs), whose Co^2+^/Co^3+^ and Fe^2+^/Fe^3+^ redox pairs mimic catalase/peroxidase activity to produce O_2_^–•^/^•^OH radicals; in vitro, these NPs achieved >80% apoptosis-mediated inhibition of MDA-MB-231 cells at 300 μg/mL. CDT efficacy is constrained by intracellular H_2_O_2_ levels and GSH overexpression, which necessitates high catalyst doses that exacerbate toxic and side effect risks. Similarly, photothermal therapy (PTT) deploys near-infrared photons (700–980 nm) absorbed by photosensitizers to generate heat, thereby inducing thermal ablation of tumor tissue through localized hyperthermia.^9^ Kampaengsri et al.^10^ engineered polyethylene glycol-encapsulated QuCy7 heptamethine cyanine NPs (QuCy7@mPEG), which absorbed >750 nm light to elevate temperatures by 24 °C (35% photothermal efficiency) under 808 nm irradiation and reduced tumor volume by 76% in chick embryo models with validated biosafety. PTT also faces challenges from poor penetration depth and uncontrolled heat diffusion, causing uneven intratumoral heating and nontargeted thermal damage.

Given the inherent limitations of monotherapies, emerging tumor-specific approaches have increasingly been incorporated into combination therapies. Representative ones include chemodynamic/photothermal therapy,^11^ photodynamic/photothermal therapy,^12^ photothermal/immunotherapy,^13^ chemodynamic/photodynamic/photothermal therapy,^14^ and chemodynamic/photodynamic/immunotherapy.^15^ Instead of merely overlapping multiple methods, these combination systems rely on synergistic interactions among therapeutic modalities to enhance antitumor efficacy while reducing drug concentrations, thereby minimizing damage to normal cells. Given the demonstrated advantages, the establishment of integrated nanotherapeutic platforms for multimodal therapy has emerged as a prevailing trend in oncology research. Within this field, nanocarriers loaded with functional small molecules have garnered more attention. Nanocarriers, benefiting from their nanoscale size (1–100 nm) and high specific surface area, provide enhanced penetration depth and a high loading capacity. The small size allows for efficient penetration through gaps in tumor vascular endothelium via the enhanced permeability and retention (EPR) effect,^16^ further facilitating passive accumulation within tumor tissue. Meanwhile, the large surface area provides abundant binding sites and pore structures that significantly increase the payload capacity for drugs and functional molecules through physical/chemical adsorption. Building on nanoscale advantages, nanocarriers can further utilize their inherent or foreign redox-active metal ions to exhibit chemodynamic effects. Functional micromolecules comprise three distinct categories based on their mechanisms: (i) direct tumoricidal agents exemplified by doxorubicin (DOX), paclitaxel, and cisplatin, which inherently exert cytotoxic effects against cancer cells; (ii) auxiliary therapeutic molecules including indocyanine green (ICG) serving as photothermal agents, chlorin e6 (Ce6) functioning as photosensitizers, and hematoporphyrin derivative (HPD) acting as sonosensitizers that harness external stimuli for therapeutic enhancement; and (iii) nanoplatform-optimizing molecules such as glucose oxidase (GOx) amplifying chemodynamic therapy efficacy, folic acid boosting endocytic efficiency, and biotin augmenting targeting specificity within delivery systems.

Nanocarriers loaded with functional molecules create advanced antitumor strategies. The rational design of nanotherapeutic platforms is essential, as they deliver multimodal tumor-targeting capabilities while amplifying efficacy through synergistic mechanisms. Consequently, systems incorporating zeolitic imidazolate framework-8 (ZIF-8), Cu^2+^ ions, and ICG&DOX dual-functional agents emerge as promising candidates. The ZIF-8 organometallic framework demonstrates high efficiency in loading molecules ranging from small compounds to biomolecular fragments through its ultrahigh surface area (1000–1800 m^2^/g) and tunable pore size (∼3.4 nm); its controllable particle size (50–200 nm), achieved by modulating the Zn^2+^/ligand ratio, optimizes the EPR effect at tumor sites while enhancing the cellular uptake efficiency.^17^ The framework degrades in acidic tumor microenvironments (pH 5.0–6.0), and the released Zn^2+^ ions contribute to increasing osmotic pressure and generate reactive oxygen species (ROS) to damage tumor cells with minimal impact on normal tissues. To overcome insufficient tumor-killing by Zn^2+^ alone, Cu^2+^ substitution exploits comparable ionic radii to trigger Fenton-like reactions, converting H_2_O_2_ into DNA-damaging hydroxyl radicals (^•^OH) while inducing mitochondrial disruption via cuproptosis.^18^ As a chemotherapeutic agent, DOX combats diverse cancers (e.g., breast and lung cancers) through three mechanisms, including DNA intercalation, free radical generation, and topoisomerase II inhibition. This broad-spectrum efficacy and critical therapeutic role have been validated by approximately 2400 global clinical trials through 2023.^19^ Concurrently, ICG, the sole NIR-I fluorophore approved by both Chinese and U.S. regulatory authorities, exhibits excitation at 750–810 nm and generates localized hyperthermia via nonradiative relaxation. Nanoencapsulation enhances its photothermal conversion efficiency (PCE) to 20%, enabling deep-tissue penetration (5–10 mm) for real-time imaging-guided therapy.^20^ All the benefits of these components exactly exploit the acidic/H_2_O_2_-rich TME and target the heat-sensitive tumor cells, inspiring the design of a novel “all-in-one” nanoplatform of Cu-doped ZIF-8 nanoparticles coloaded with ICG and DOX (Cu-ZIF-8@ICG&DOX, CZID) for combined CDT/PTT/chemotherapy. In this system, beyond their intrinsic functions, the localized heat generated from ICG under irradiation is designed to accelerate the degradation of the ZIF-8 framework, further inducing a promoted release of both Cu^2+^ ions and DOX, finally achieving superior chemodynamic and chemotherapeutic efficacy through photothermal-enhanced synergism.

To our limited knowledge, such a novel CZID nanoplatform has rarely been reported; more importantly, the synergistic mechanism of leveraging PTT to enhance both CDT and chemotherapy at the cellular level remains underexplored. As shown in Scheme 1, the rationale, fabrication, and anticipated mechanism of the CZID nanoplatform were outlined. This study began with the synthesis and optimization of Cu-doped ZIF-8, followed by the coloading of ICG and DOX to construct the final CZID formulation. Beyond its structural characterization and physicochemical analysis, the proposed therapeutic strategy, particularly the photothermal-triggered synergistic effect leading to tumor cell apoptosis, was further validated through cellular uptake studies, aiming to achieve a promising efficacy–safety profile for multimodal antitumor therapy.

**Scheme 1:**
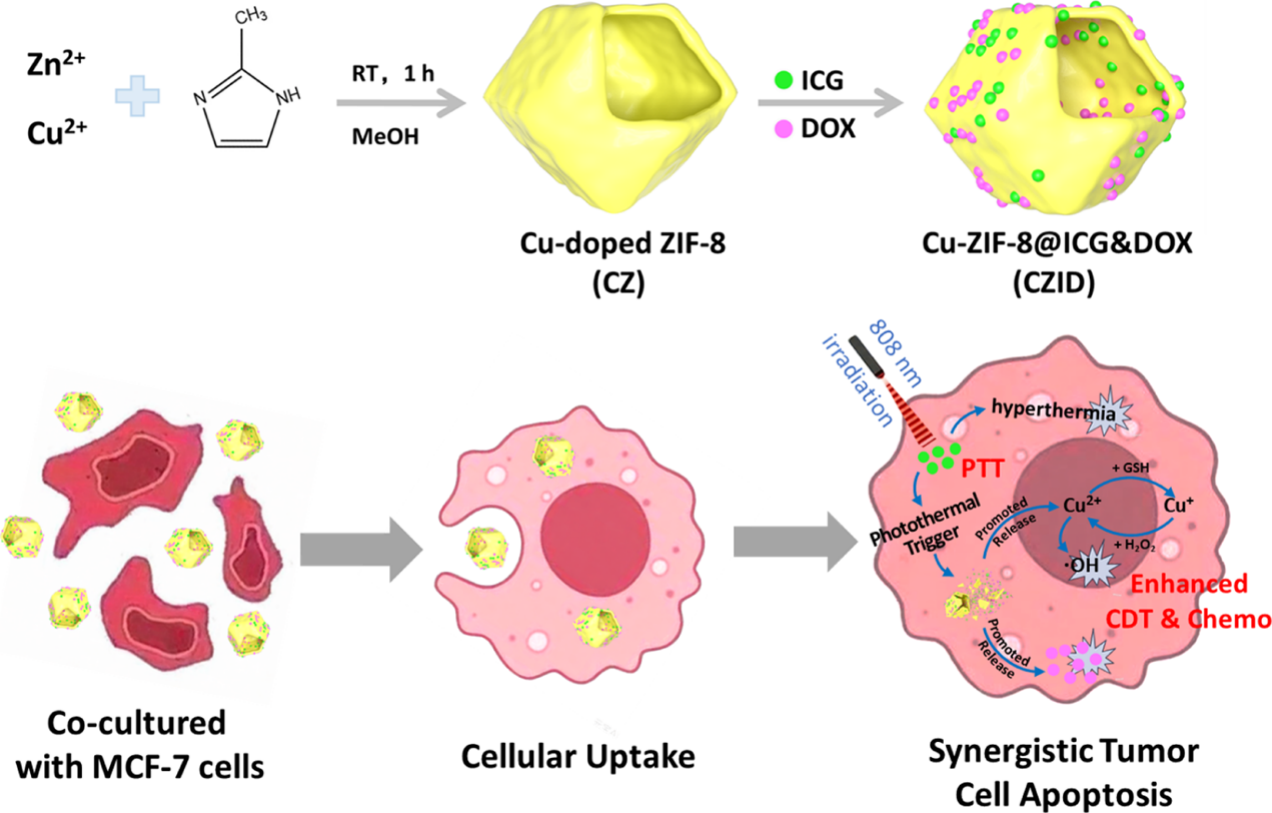
Synthesis Route of the CZID Nanoplatform and Its Proposed Mechanism for Synergistic Tumor Therapy^a^

a Source: created by the authors.

## 2 Experiments

### 2.1 Materials

2-Methylimidazole, Zn­(NO_3_)_2_·6H_2_O, Cu­(NO_3_)_2_·3H_2_O, reduced glutathione (GSH), and 5,5′-Dithiobis­(2-nitrobenzoic acid) (DTNB) were purchased from Aladdin Bio-Chem Technology Co., Ltd. Methanol, indocyanine green (ICG), and doxorubicin (DOX) were obtained from Macklin Biochemical Co. Ltd. All reagents were used as received without further purification.

Phosphate-buffered saline (PBS) was purchased from Fuzhou Scientific Phygene Biotechnology Co. 4′,6-Diamidino-2-phenylindole (DAPI), 2′,7′-dichlorofluorescin diacetate (DCFH-DA), calcein-AM, and propidium iodide (PI) were obtained from Aladdin Biochemical Technology Co., Ltd. DMEM, fetal bovine serum (FBS), and cell counting kit-8 (CCK-8) were purchased from Gibco Life Technologies. MCF-7 breast cancer cells, 4T1 mouse breast cancer cells, and human normal breast epithelial cells of MCF-10A were obtained from the Cell Bank of the Chinese Academy of Sciences, located in Kunming.

### 2.2 Synthesis of Cu-Doped ZIF-8 NPs

(1.25 – *x*) mmol Zn­(NO_3_)_2_·6H_2_O and *x* mmol Cu­(NO_3_)_2_·3H_2_O were sequentially added in 12 mL of methanol for preparing solution A, with a default Cu doping level of *x* ranging from 0 to 0.375 at an interval of 0.125. 10 mmol methylimidazole was dissolved in 20 mL of methanol as solution B. The reaction solution was obtained by mixing both the A and B solutions; after full reaction under stirring for 1 h at ambient temperature, the reddish-brown precipitates appeared, which were then treated with centrifugation, washing with methanol, and drying at 60 °C to receive the final product of Cu-doped ZIF-8 NPs (Cu-ZIF-8, CZ).

### 2.3 Synthesis of the Cu-ZIF-8@ICG&DOX Nanoplatform

CZ NPs were redispersed in 30 mL of methanol and then slowly added with 10 mL of ICG and DOX solution; both were 4 mg/mL by using methanol as the solvent. The mixed suspension was further stirred for at least 3 h in the dark until its color turned purple. The newly formed precipitates were received as Cu-ZIF-8 NPs dual-loaded with ICG and DOX (Cu-ZIF-8@ICG&DOX, CZID nanoplatform) after undergoing the same separation operations as those for the nanoparticle preparation in Section 2.2.

Accordingly, Cu-ZIF-8 single-loaded with ICG (Cu-ZIF-8@ICG, CZI) and Cu-ZIF-8 single-loaded with DOX (Cu-ZIF-8@DOX, CZI) as two controls were also synthesized following the same procedure and parameters of CZID.

### 2.4 Characterizations

The synthesized products were characterized by the X-ray diffractometer (XRD, Rigaku Ultima IV, Japan) and the transmission electron micrographs (TEM, Thermo Scientific Talos F200S G2, USA) combined with an energy-dispersive X-ray detector (EDS, Super-X) and X-ray photoelectron spectroscopy (XPS, Thermo Scientific ESCALAB 250Xi, USA). The actual substitutions of Cu ions on Zn sites are quantified by inductively coupled plasma mass spectrometry (ICP-MS, PerkinElmer ICP 2100, USA). The UV–vis spectra of the received products were recorded under a UV–vis spectrophotometer (TU-1901, PERSEE, China), and their specific surface area and pore information were determined by the surface area and porosity analyzer (BET, Micromeritics ASAP2460, USA). Zeta potentials combined with size distributions of ZIF-8, CZ, and CZID were measured on a zeta potential meter (Zetasizer Nano ZS90, Malvern, Britain) using deionized water as the dispersant.

### 2.5 Photothermal Performance Determination

CZID suspensions with designated concentrations of 50–1000 μg/mL were monitored for temperature rise under 1 W/cm^2^ of NIR laser irradiation at 808 nm for 10 min. The one with obvious temperature-rising was further adopted to figure out the changes at different powers from 0.8 to 1.2 W/cm^2^ and also subjected to the stability test of three-cycle on/off irradiation. All photothermal measurements were performed with deionized water as a control. From these tests, the photothermal conversion efficiency (η) was calculated based on the temperature increases of the system relative to pure water under laser irradiation, following the method reported in ref 21 The calculation is derived from the energy balance principle, which accounts for the heat dissipation difference between the sample and the solvent, and the detailed formulas are provided in Section S1 of the Supporting Information.

### 2.6 GSH Depletion

200 μL portion of 200 μg/mL CZID suspension and 200 μL of 2 mM GSH aqueous solution were mixed, and a total of 400 μL of the mixture was separately added to 2.5 mL of PBS solution with a varied pH of 7.4, 6.5, or 5.0. The reaction was allowed to stand for some time (0, 1, 3, 6, and 12/24 h), followed by the addition of 100 μL of ethanol containing 1 mg/mL DTNB. After that, the UV–vis spectrum for the resulting TNB product of the mixture was recorded, and the absorbance at 412 nm was determined as a function of the appointed reaction times.

### 2.7 Ion and Drug Release Determination

30 mg of CZID was dispersed in 90 mL of 0.1 M PBS at pH 5.0 or 7.4. The suspension was then shaken at 150 rpm and 37 °C. At designed time points of 1, 3, 6, 12, and 24 h, the suspension was withdrawn and filtered through 0.22 μm filters. The concentrations of released Cu^2+^ ions in the filtrate were quantified by ICP-MS (PerkinElmer ICP 2100, USA), and the contents of ICG and DOX were determined by measuring their absorbance using the UV–vis spectrophotometer (TU-1901, PERSEE, China) and calculating via standard curves in Figure S2. To evaluate the effect of temperature on the release behavior, the same amount of CZID was dispersed in the isometric pH 5.0 PBS and shaken at 150 rpm and different temperatures (25, 35, 45, and 55 °C). Samples were collected at the same time points and analyzed as described above. All experiments were repeated 3×.

### 2.8 Cellular Staining and Viability Assessment

#### 2.8.1 In Vitro Cellular Uptake

After experiencing normal incubation under the same culture conditions overnight, the MCF-7 cells with a density of 1 × 10^5^/dish continued to be incubated in the alternative CZID-contained medium with 50 μg/mL for another 3 h, rinsed 3× with sterile PBS, and stained with DAPI at 1 mg/mL for 10 min in the dark. Whether the cellular uptake of nanoproducts occurred was verified on the same CLSM. In detail, the DAPI and DOX were excited by the 405 nm laser and the 559 nm laser, respectively.

#### 2.8.2 Intracellular ROS Detection

For intracellular ROS detection, MCF-7 cells were treated with one of the following nanoplatforms at 50 μg/mL: CDI, CZD, or CZID. After 3 h of coculture following overnight incubation, the cells were rinsed with PBS, stained in the dark, and imaged under CLSM. The differences were the addition of one new step of 1 W/cm^2^ NIR laser irradiation at 808 nm for 10 min before the coculturing, and the adoption of 10 μM DCFH-DA as the ROS kit and 50 mg/mL Rosup as the positive control, with the staining time of 30 min. DCF excitation was performed by the 488/525 nm laser.

#### 2.8.3 Live/Dead Cell Staining

MCF-7 cells were seeded in 6-well plates with a density of 1 × 10^5^/well and incubated at 37 °C under a 5% CO_2_ atmosphere for 24 h. The culture medium was pipetted out, and fresh medium containing each CZI, CZD, or CZID was added. MCF-7 cells surrounded by each nanoplatform were cocultured for another 24 h under the same conditions, rinsed with sterile PBS to remove residual serum, and then stained with the mixture of Calcein-AM and PI for 15 min in the dark. The morphology observation for stained MCF-7 cells was performed on a confocal laser scanning microscope (CLSM, IX73, OLYMPUS, Japan).

#### 2.8.4 In Vitro Cell Viability

MCF-7 tumor cells and MCF-10A normal cells were chosen as model cells to verify their viability by coculturing with 3 synthesized nanoplatforms. Each cell in logarithmic growth was seeded into a 96-well plate at 8 × 10^3^/well and incubated at 37 °C under a 5% CO_2_ atmosphere. The suspension of CZI, CZD, or CZID was diluted to the working concentration with the culture medium and then UV-irradiated for 30 min for sterilization. After cell attachment, the medium in each well was replaced by an equal volume of diluted suspension and another 24 h of cell incubation under the same conditions proceeded. Then, 100 μL/well medium containing 10% of the CCK-8 kit was added, and the mixture was incubated for 2 h. The absorbance of each target well was determined on a microplate reader (SPARK 10 M, TECAN, China) at 450 nm. The cell viabilities of the samples were calculated by the formula below:

1 $$Cell\;viability=\frac{{OD}_{sample}-{OD}_{blank}}{{OD}_{control}-{OD}_{blank}}\times 100\%$$

where OD_sample_, OD_Control_, and OD_Blank_ represented the absorbance of the experiment, the control, and the blank, respectively. In particular, NIR groups were established to evaluate the enhanced antitumor efficacy of the nanoplatforms under external irradiation. The procedure followed the aforementioned coculture protocol, with one modification: during the 24 h incubation of MCF-7 cells with suspensions of CZI, CZD, or CZID, a 10 min, 808 nm laser irradiation at a power density of 1.0 W/cm^2^ was administered at the 12 h point. The cytotoxicity level was determined based on criteria specified in ISO 10993-5:2009.^22^

In addition, 4T1 cells were employed in the cell viability assay to evaluate the broad-spectrum antitumor efficacy of CZID, following the same experimental procedure as that described for MCF-7 cells.

### 2.9 Statistical Analysis

Data from repeated experiments are expressed as mean ± standard deviation (SD), with error bars in each graph representing the SD. Statistical significance was analyzed by one-way ANOVA with Tukey’s test, using asterisks to denote significance levels: *p < 0.05, **p < 0.01, and ***p < 0.001.

## 3 Results and Discussion

### 3.1 Characterization Results Analysis

#### 3.1.1 Optimization of the Cu Doping Content in ZIF-8

Figure 1 displays the phases, actual Cu^2+^ molar contents, and morphologies of the CZ nanoparticles. In Figure 1a, the XRD patterns reflected that the diffraction peaks at about 7.3°, 10.3°, 12.7°, 14.6°, 18.8°, 22.1°, 24.2°, and 26.8° in 2θ corresponded to the (011), (002), (112), (022), (222), (114), (233), and (134) crystal planes belonging to ZIF-8, respectively. Cu doping still maintained the samples with the ZIF-8 phase but caused the peak positions to shift toward the large angle direction owing to its ionic radius of 0.72 Å, smaller than that (0.74 Å) of Zn, and increasing the Cu doping content made such a shifting more obvious, especially for the peak of the (011) plane. In Figure 1b, the actual Cu^2+^/Zn^2+^ molar ratio given by ICP tended to increase and then decrease, with a maximum value of 7.46%. It was inferred that there was an upper limit for the CZ since the binding affinity of Cu^2+^ ions to the 2-methylimidazole ligand was lower than that of Zn^2+^ ions to one.^23^ In Figure 1c, the lower Cu^2+^ molar content in ZIF-8, such as 10%, did not change the typical ZIF-8 morphology, the characteristic of rhombic dodecahedra on its related nanoparticle sample. As it increased to 30%, the corners at the rhombohedron edges vanished; the newly formed arc enabled the 30Cu-ZIF-8 nanoparticles to present a spheroidal shape. The size statistics over not less than 100 particles for each CZ nanoparticle were found to be normally distributed, showing good size uniformity, and the doping content increasing from 10% to 30% caused the average particle size to expand to 140.5 nm from 91.7 nm; such a change occurred because the weaker binding affinity to the ligand from Cu^2+^ ions slowed down the deprotonation process in ZIF-8 generation, further suppressing the nucleation rate and accelerating nucleus growth. To enhance the penetration and retention of the CZ at the tumor site, the particle size should be controlled below 100 nm. To achieve an efficient Fenton-like reaction in chemodynamic therapy, the crucial Cu^2+^ in ZIF-8 must be maintained at a higher content level. Both conditions optimized the Cu^2+^/Zn^2+^ molar ratio to 25%, and the 25Cu-ZIF-8 nanoparticles with an average size of 99.6 nm acted as the target for subsequent double loading. Figure 1d displays the element detection of 25Cu-ZIF-8 nanoparticles under EDS. It was seen that Zn, C, and N elements established the whole organic framework, and the doped Cu element was uniformly distributed over the whole nanoparticle.

**Figure 1:**
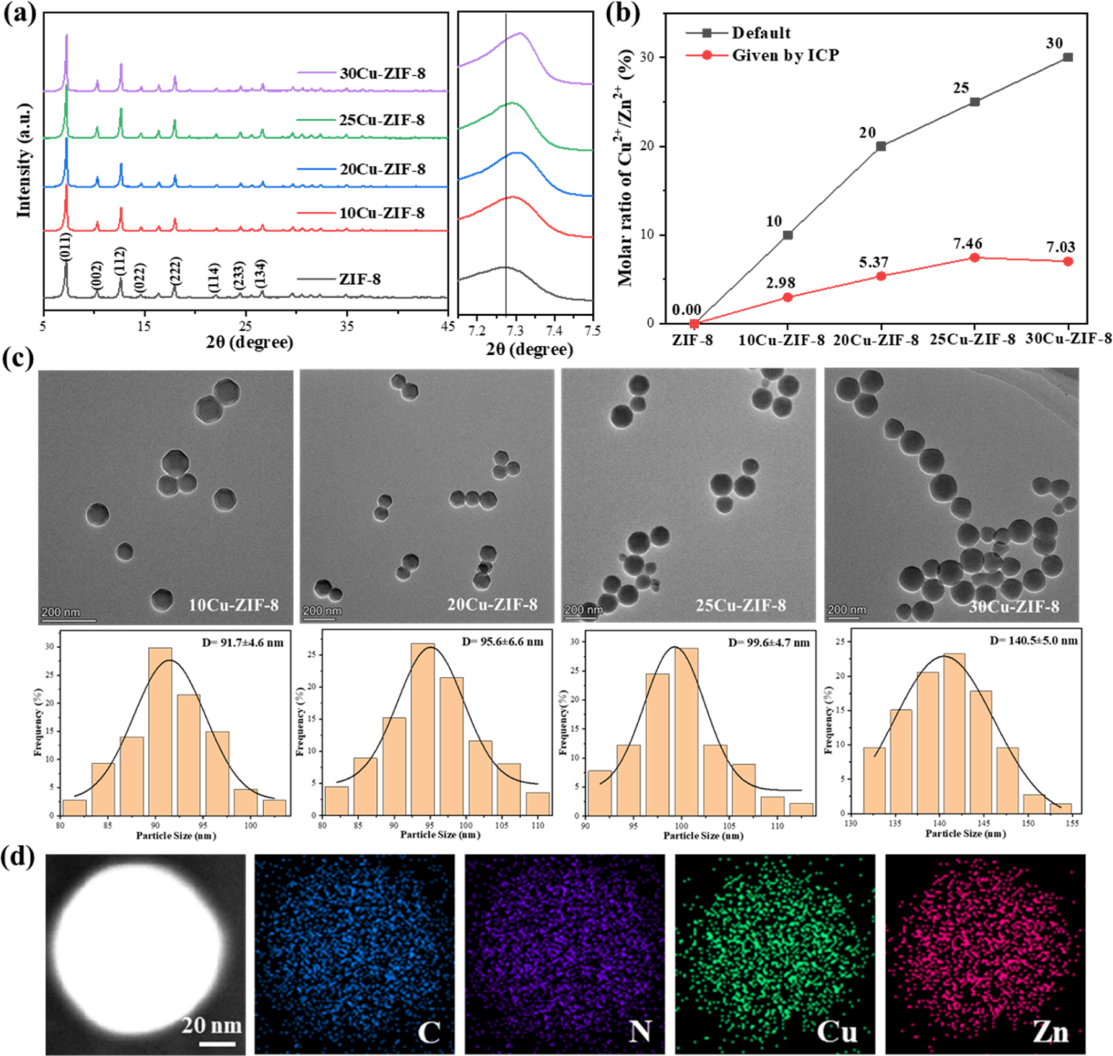
Characterizations for optimizing the Cu^2+^/Zn^2+^ molar ratios: (a) XRD patterns; (b) actual Cu^2+^ doping contents in ZIF-8 determined by ICP; (c) TEM images and resulting particle size statistics; and (d) element distributions within a single nanoparticle.

#### 3.1.2 Structure of Cu-ZIF-8 Dual-Loaded with ICG and DOX

Whether both ICG and DOX succeeded in being loaded on 25Cu-ZIF-8 nanoparticles is summarized in Figure 2. The XRD pattern in Figure 2a showed that these two micromolecules caused no shift to the diffraction peak positions of CZ but lowered the peak intensities; such a change was attributed to the shielding effect from the surface ICG and DOX acting on the inner CZ. This phenomenon was commonly observed in core–shell materials like Fe_3_O_4_@C@MnO_2_,^24^ where interfacial contact dominated the compositing process. Figure 2b shows the XPS high-resolution spectra of Cu 2p; the differentiating/fitting gave two peaks centered at 932.94 and 953.06 eV that corresponded to Cu 2p3/2 and Cu 2p1/2 originated from Cu^2+^, and the characteristic satellite peak belonging to Cu^2+^ was also found at ∼945.48 eV. Both results proved that Cu continued to carry +2 valence in ZIF-8 after the ICG&DOX loading, and its role of generating Fenton-like responses in the tumor microenvironment was still guaranteed. It was seen from the TEM image of Figure 2c that the CZID transformed into a spherelike morphology with the disappearance of the original rhombic dodecahedron feature of ZIF-8, and a carbon coating was observed on the surface. This structural evolution originated from the adsorption of ICG and DOX by electrostatic interactions with the imidazole moiety of ZIF-8,^25^ resulting in a core–shell structure with CZ as the core and ICG&DOX as the shell. In addition, the loading of micromolecules under long-term stirring partially disrupted the good dispersity of CZ, leading to worsening nanoparticle agglomeration and an expansion in the size distribution of CZID, as shown in the DLS of Figure 2d. Notably, the increase in the PDI from 0.092 for CZ to 0.219 for CZID also reflected the size distribution broadening. However, the value of 0.219 is considered low based on established criteria in colloid science,^26^ indicating that the CZID nanoplatform exhibited a narrow size distribution, which was essential for maintaining a stable colloidal suspension. Zeta potentials variation from ZIF-8 to CZ and then to CZID was illustrated in Figure 2e; the product surface changed its positively charged state (+20.3 mV, +21.3 mV) into an opposite one (−14.4 mV) after the drug double-loading, and it was the result of negatively charged micromolecules being attracted by CZ under electrostatic interaction^27^ and then being chemically bonded with the organic framework under coordination, which also indirectly verified that Cu^2+^ doping aimed to occupy the Zn^2+^ position. In Figure 2f, both isothermal adsorption–desorption curves exhibited the characteristic of a Type I isotherm; CZ and CZID could be identified as microporous adsorbents with calculated pore sizes of 1.88 and 1.90 nm and specific surface areas of 1946.2 m^2^/g and 1819.5 m^2^/g, respectively. In contrast to the almost constant pore size, the specific surface area declined as ICG and DOX succeeded in being loaded on the CZ. These micromolecules attaching to the MOF normally contributed to increasing the surface roughness and further offered more surface sites, while such an opposite trend indicated the decline was affected by the agglomeration to a greater extent, as supported by the TEM results in Figure 2c.

**Figure 2:**
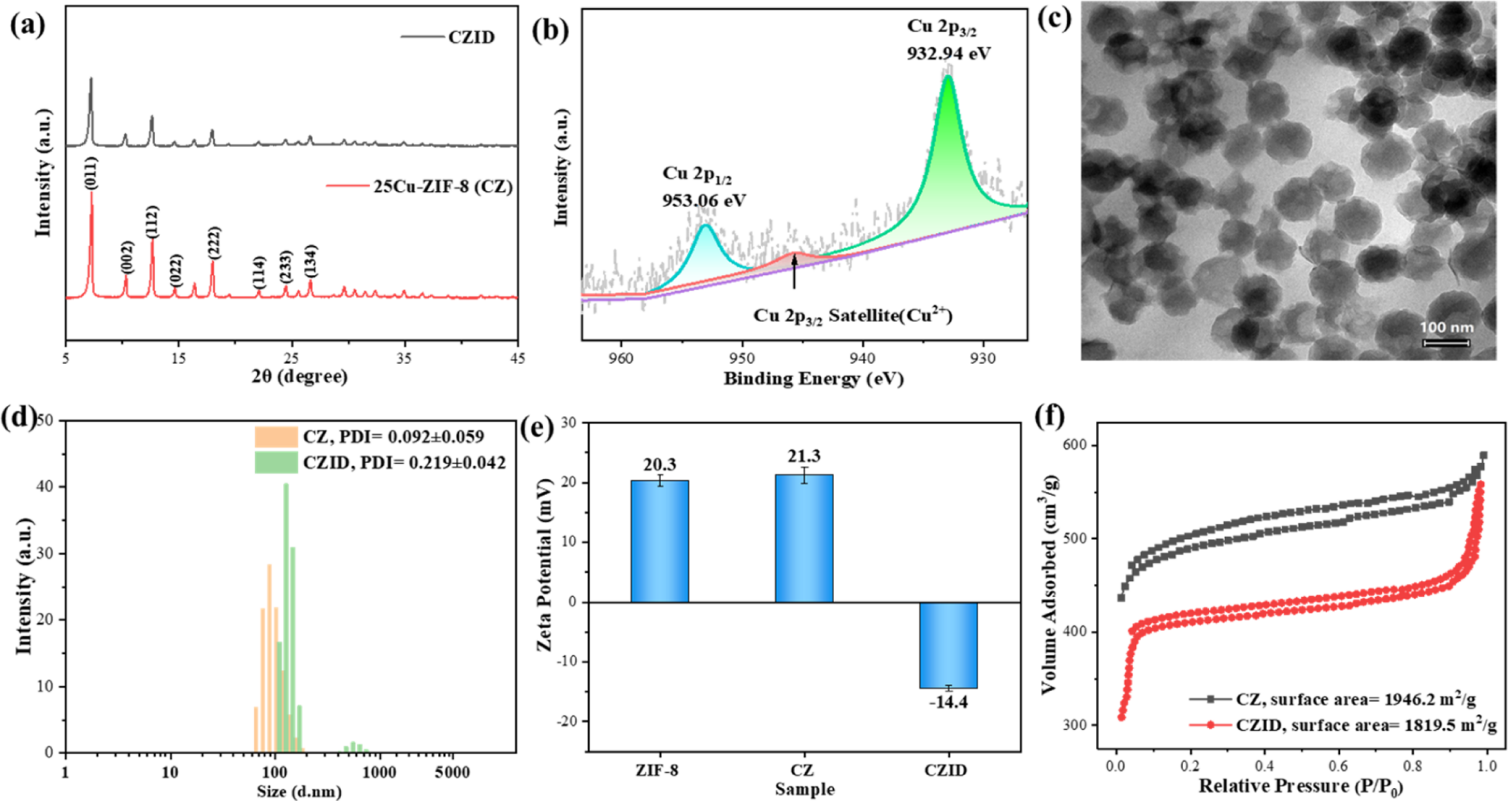
Characterizations for double loading ICG&DOX on CZ: (a) XRD patterns; (b) XPS spectra of Cu element; (c) TEM morphology; (d) particle size distribution under DLS measurement; (e) surface zeta potentials comparison, error bars: SD; and (f) N_2_ isothermal adsorption–desorption curves of CZ and CZID.

#### 3.1.3 Determination of Loading Contents in CZID

When redispersed in deionized water, nanoproducts exhibited color change, as shown inFigure S1, which also confirmed the loading of micromolecules succeeded visually, since the double-loaded CZID exhibited a violet color that mixed with the green from ICG-loaded CZI and the light magenta from DOX-loaded CZD and was different from the transparent ZIF-8 and CZ. The resulting UV–vis spectra based on these suspensions are recorded in Figure 3a; no peak was detected on CZ in the measured wavelength range, indicating that all UV–vis absorption was triggered by micromolecules. Thus, the peak only appearing on CZI or CZD belonged to the characteristic absorption of ICG and DOX, respectively, and CZID owned both. Compared to the standard solutions provided inFigure S2a, ICG and DOX in CZID changed their absorption positions to ∼502 and ∼825 nm. The bathochromic shift happening in both was due to the coordination interaction between the micromolecules and the organic framework.^28^ Moreover, the difference in absorbance also suggested that the loading content of DOX was higher than that of ICG in CZID. Figure 3b shows the loading contents of ICG and DOX in CZID under increased concentrations to pursue the optimal result. Both loading contents exhibited a trend of first increase and then decrease, reaching their top values at 4 mg/mL. Following the absorbance–concentration relation curves given inFigure S2b, the fitted linear equations yielded the maximum capacities for ICG and DOX in CZID, which were 23.01 μg/mg and 122.43 μg/mg, respectively, and the total loading ratio of the two micromolecules was calculated to be 14.54%.

**Figure 3:**
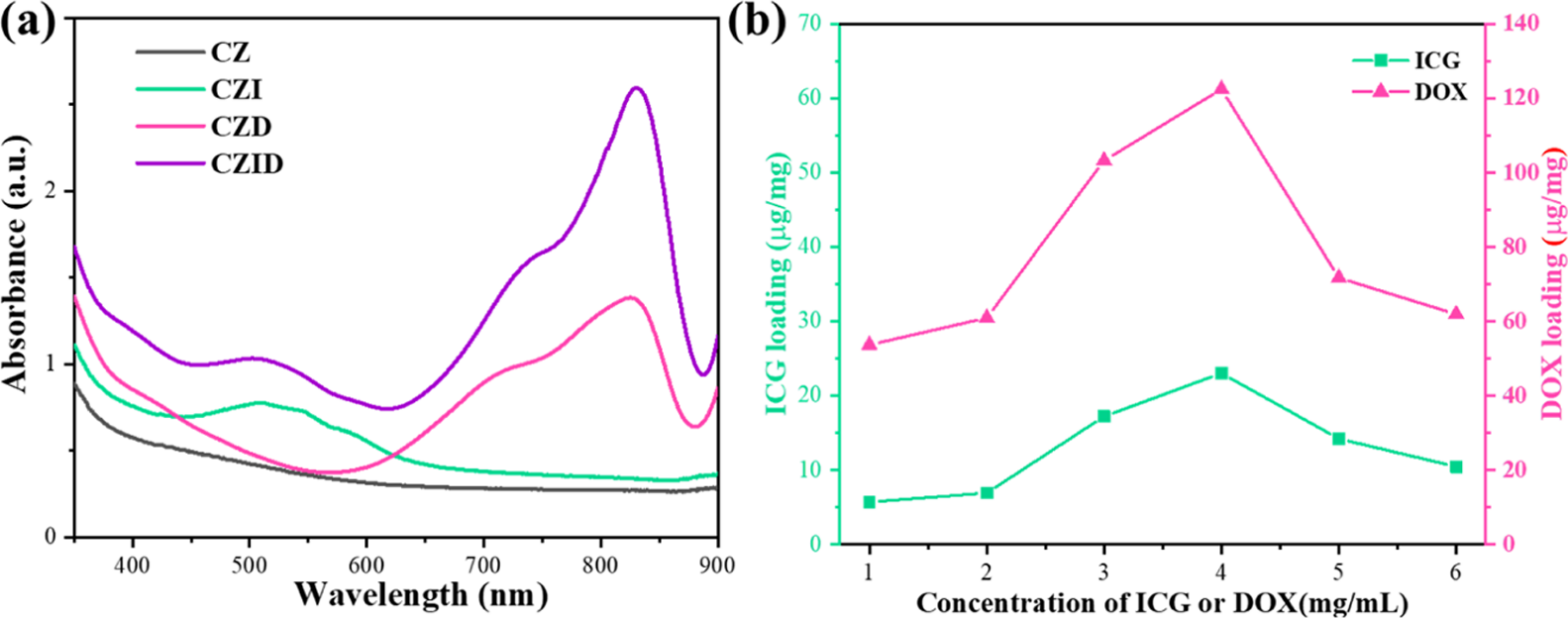
UV–vis absorption spectra (a) of synthesized products and ICG and DOX loading contents (b) determined in CZID.

### 3.2 Physicochemical Properties Analysis

#### 3.2.1 Photothermal Performance

The photothermal performance of CZID when irradiated by an 808 nm NIR laser is shown in Figure 4. It was found that the temperature rise of CZID has positive correlations with the suspension concentration and the power density. As shown in Figure 4a, the dramatic rise in warming started at the concentration of 200 μg/mL, and 10 min of irradiation enabled the temperature to reach 46.7 °C and above. Compared to the water control, the vast majority of irradiation warming came from the CZID platform, with only ICG acting as the photothermal donor; the warming effectiveness appeared to be concentration-dependent but was controlled by the loading content. Similarly, increasing the power density to 1.2 W/cm^2^ could also bring the temperature up to 48.3 °C, but the amplitude was not as obvious as that induced by the suspension concentration in Figure 4b. Taking the concentration of 200 μg/mL combined with the power density of 1 W/cm^2^ for example, the PCE of CZID after 10 min of irradiation was calculated as 38.2%. Under the on/off irradiation mode, as shown in Figure 4c, the CZID suspension displayed little variation in peak temperature over 3 cycles, especially since the decline range was smaller than that of the ICG aqueous solution, which indicated CZID had good photothermal stability. Because of the above, the temperature is raised to about 45 °C to avoid abnormal expression of heat shock protein (HSP) by cancer cells due to heat and, at the same time, maximize the effect of photothermal therapy to achieve apoptosis of tumor cells without causing irreversible damage to normal cells in the tumor microenvironment^29^ and also transiently increase vascular permeability, thereby facilitating better uptake and accumulation of CZID by tumor cells;^30^ coupled with PCE and stability, this nanoplatform was suitable to be applied in PTT.

**Figure 4:**
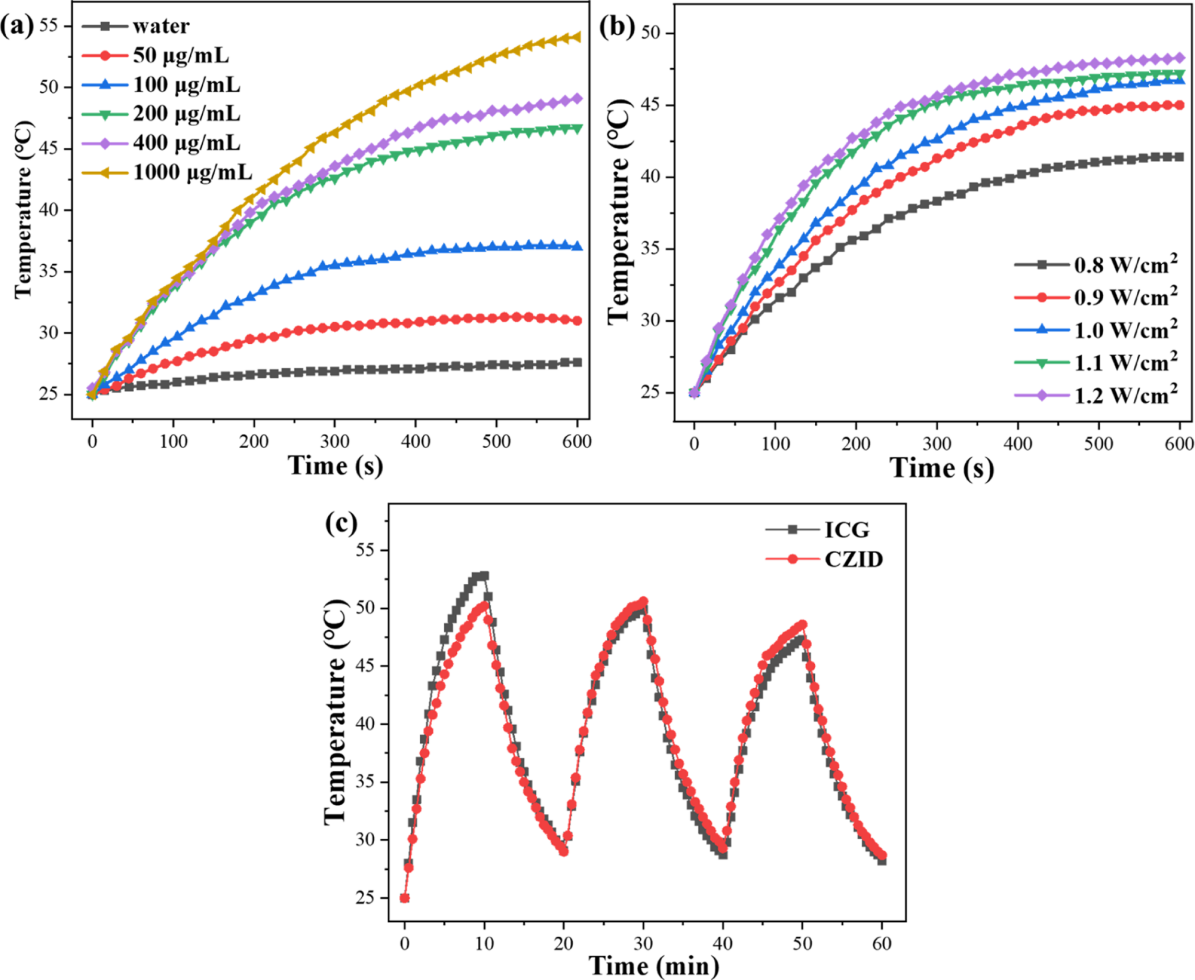
Photothermal performances of CZID under 808 nm laser irradiation: (a) temperature rising curves at different suspension concentrations (1 W/cm^2^); (b) temperature rising curves at different irradiation power (200 μg/mL); (c) thermal cycling behaviors under on/off irradiation for 3 times.

#### 3.2.2 Chemodynamic Performance Expressed in GSH Depletion

Because GSH lacks significant UV absorption, its depletion by CZID could not be monitored directly. Therefore, it was measured indirectly via a follow-up reaction with DTNB, which produced the strongly absorbing product TNB. As shown in Figure 5a–c, the peak at ∼410 nm was attributed to the characteristic absorption of TNB, and its difference in absorbance could be utilized to report the changes in GSH residue. Following an increased reaction time between GSH and CZID, the absorbance of TNB gradually decreased regardless of pH conditions of 7.4, 6.5, or 5.0, indirectly reflecting that less GSH remained. Although the depletion of CZID to GSH exhibited the same change trend in all 3 pH environments, it differed in efficiency. The sharp decrease in TNB absorbance that happened in the slightly acidic environments took half of the time than that happened in the slightly alkaline one; especially, over 80% of GSH was depleted by CZID under the condition of pH 5.0, and profound GSH depletion inactivated glutathione peroxidase 4 (GPX4), causing lipid peroxide accumulation that initiated Fenton-like oxidative chain reactions, disrupting membrane integrity to kill tumor cells.^31^

**Figure 5:**
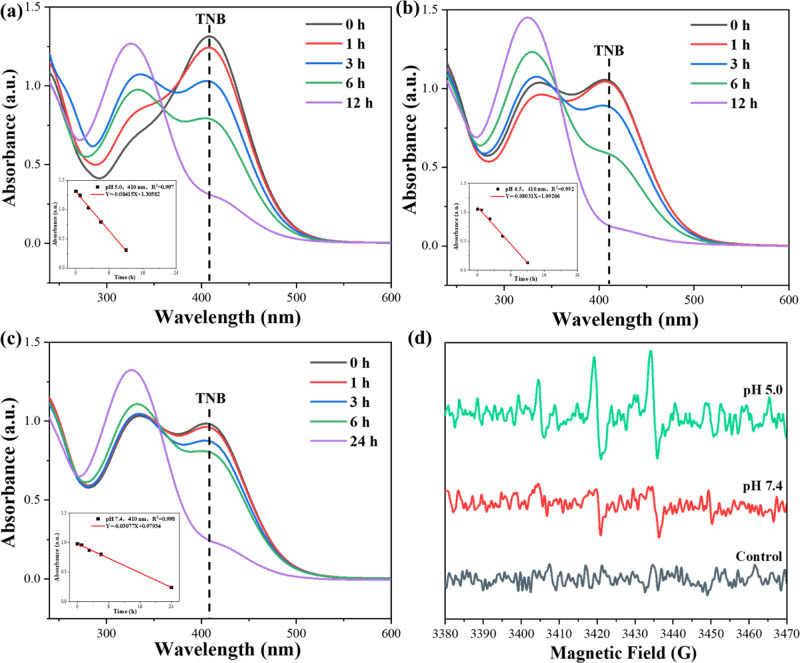
GSH depletion of CZID expressed in UV–vis absorbance changes of resulting TNB over time under varied pH environments: (a) pH 5.0, (b) pH 6.5, and (c) pH 7.4. (d) EPR spectrum of CZID for determining ROS.

Moreover, the insets in Figure 5a–c disclosed that GSH depletion varied linearly with time, and the absolute values of the slopes in the fitted equations provided a ranking on pH condition of 5.0 ≈ 6.5 > 7.4, indicating CZID carried an evident pH-responsive profile on GSH depletion. The fundamental constituent of CZID stands for ZIF-8, a kind of MOF that is more prone to structural collapse in acidic environments, causing more Cu^2+^ ions to be released. As the source for GSH depletion, more Cu^2+^ ions being reduced into Cu^+^ ions required the consumption of more GSH; thus, the acidic condition fitted CZID to play its advantages on triggering the subsequent Fenton-like reaction.^32^ After completion of the reaction of GSH with CZID, the product with exogenetic H_2_O_2_ was ready for detecting the generated reactive oxygen species (ROS), and the obtained EPR spectrum is displayed in Figure 5d. It was seen that there was electromagnetic resonance signals detected at both pH conditions of 7.4 and 5.0, as compared to the control without CZID. The generation of the hydroxyl radical (^•^OH) was confirmed, as evidenced by the characteristic 1:2:2:1 quartet signal in the EPR spectrum, which originates from the Fenton-like reaction between Cu^+^ and H_2_O_2_ (Cu^+^ + H_2_O_2_ → Cu^2+^ + ^•^OH + OH^−^).^33^ Owing to the difference in signal intensity, a higher amount of ^•^OH radicals was produced at pH 5.0 compared to those produced at pH 7.4, indicating CZID in the acidic environment could accelerate the generation of free radicals, providing a better chemodynamic performance.^34^

#### 3.2.3 Ion and Drug Release Profiles under Varied Conditions

The cumulative release profiles of Cu^2+^ ions, DOX, and ICG from the CZID nanoplatform under varied physiological conditions are shown in Figure 6. Figure 6a reveals that all three mentioned components exhibited an initial burst release within the first few hours, followed by a sustained and controlled release phase over 25 h. Notably, their release kinetics were strongly pH-dependent, with a significantly faster and greater cumulative release at pH 5.0 than at pH 7.4. Such a distinct contrast was attributed to the acid-triggered degradation of the ZIF-8 framework, a key finding that aligned with the GSH depletion demonstrated in Figure 5. Furthermore, as shown in Figure 6b, the release kinetics of all components were positively correlated with temperature, accelerating as the temperature increased from 25 to 55 °C, simulating the photothermal effect of ICG under laser irradiation. The dual-responsive release characteristics of CZID with respect to both acidic pH and elevated temperature, as demonstrated by the release profiles, were crucial for achieving synergistic antitumor efficacy. Once the tumor microenvironment was reached, the rapid release of Cu^2+^ ions not only depleted glutathione (GSH) but also initiated a Fenton-like reaction to generate highly toxic hydroxyl radicals (^•^OH), thereby enabling CDT. Meanwhile, the photothermal effect under NIR irradiation accelerated the release of two drugs. The generated hyperthermia from ICG directly killed tumor cells, while the enhanced release of DOX contributed to ROS generation. These processes could establish a self-reinforcing cycle that synergizes chemodynamics, photothermal processes, and chemotherapy.

**Figure 6:**
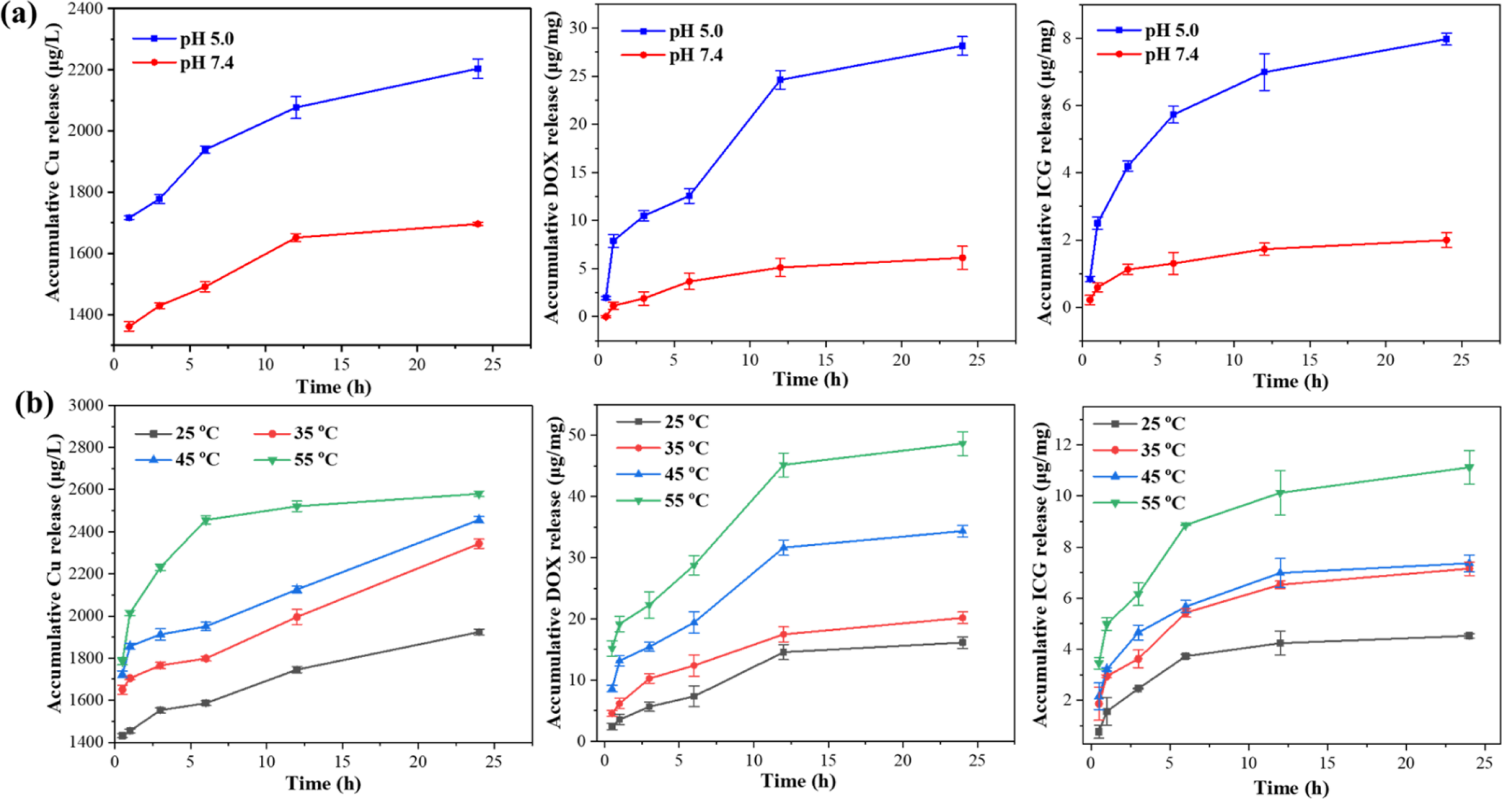
Cumulative release profiles of Cu ions, DOX, and ICG (from left to right) from CZID in PBS under different conditions: (a) at pH 5.0 and 7.4 at 25 °C; (b) at varying temperatures at pH 5.0. Error bars: SD.

### 3.3 In vitro Cell-Related Results Analysis

#### 3.3.1 Cellular Uptake and Intracellular ROS

Taking MCF-7 as tumor model cells, whether the cellular uptake could occur on CZID is displayed in the CLSM images of Figure 7. After 3 h coculturing, DAPI succeeded in staining the nuclei of MCF-7 cells, and these blue fluorescent nuclei, differing in sizes and numbers, also confirmed that the model cells as cancerous.^35^ Similarly, under external laser excitation, the DOX drug emitted red fluorescence, which appeared in the MCF-7 cell coculture group with CZID but not in the control group. In the merged CLM images, blue nuclei were also observed to be surrounded by red DOX, and such a large amount excluded its origin as free DOX, suggesting that CZID was capable of being endocytosed by cells and the loaded ICG or DOX could be further released into the tumor microenvironment for its respective photothermal and chemotherapeutic functions. In addition, the 3 h of cellular uptake time for CZID was far below that of 6 h for some phenolic nanomaterials,^36^ equipping CZID with a more competitive efficiency.

**Figure 7:**
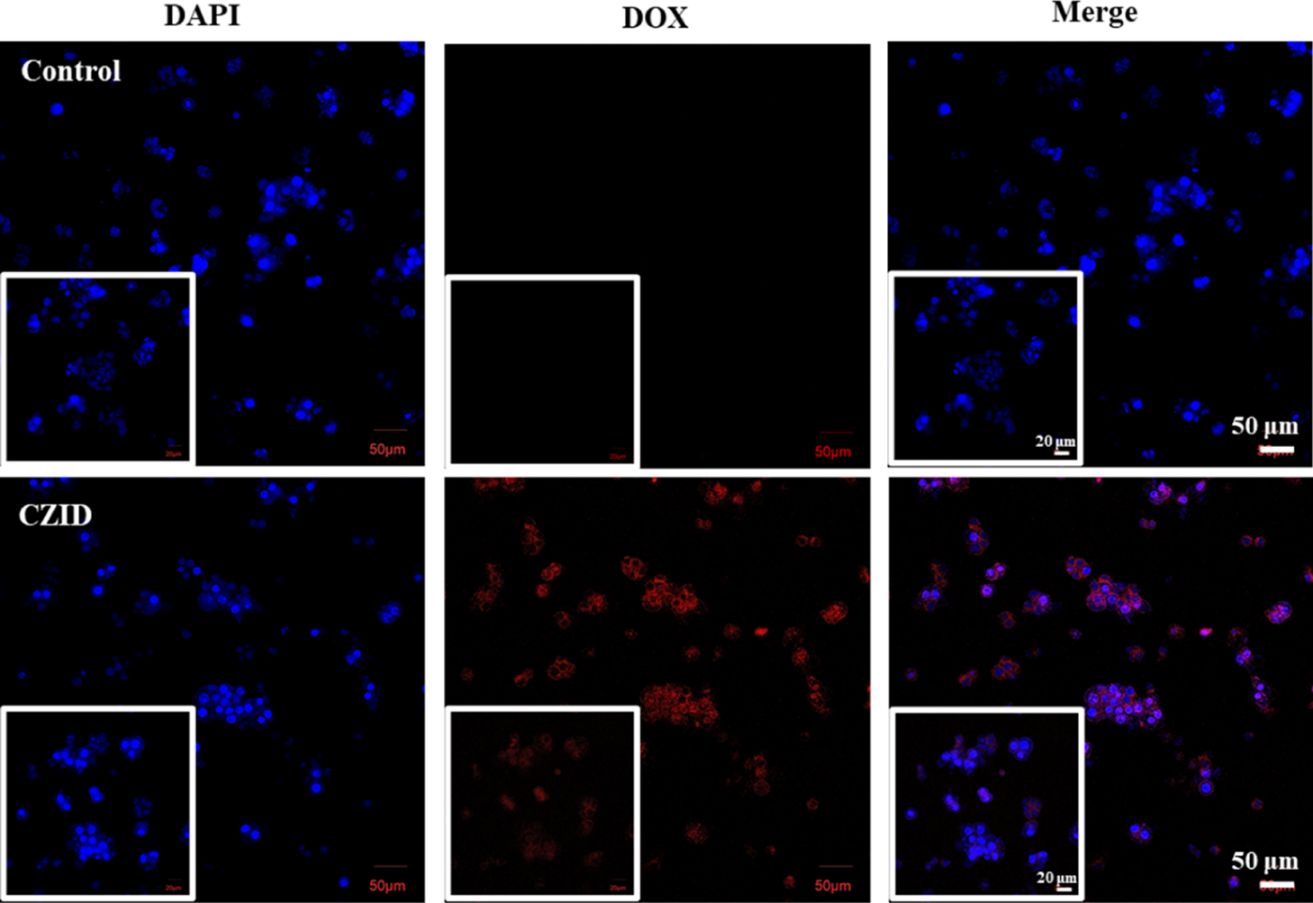
CLSM images of MCF-7 cells after 3 h coculturing with CZID (50 μg/mL), with a 400× magnified view as inset.

#### 3.3.2 Intracellular ROS and Live/Dead Cell Staining on Tumor Cells

Owing to the cellular uptake, the generation of ROS by the CZ-based nanosystems in MCF-7 cells became traceable. Cells stained with DCFH-DA emitted green fluorescence upon ROS generation, as displayed in Figure 8 under 488/525 nm excitation. In the absence of 808 nm NIR irradiation, the fluorescence intensity observed in Figure 8a across the groups followed the order: CZID ≈ CZD > CZI > Control. This trend was attributed to the distinct ROS generation mechanisms. The superior performance of both CZID and CZD stemmed from the combined effect: (1) the released Cu^2+^ ions from the acid-degraded ZIF-8 framework participated in an intracellular Fenton-like reaction, consuming glutathione (GSH) to generate highly oxidative ^•^OH radicals; and (2) the concurrently released chemotherapeutic drug DOX contributed to the ROS signal by inducing superoxide anion (^•^O_2_^–^) generation via mitochondrial disruption, and these anions were rapidly converted to hydrogen peroxide (H_2_O_2_), thereby intensifying the fluorescence.^37,38^ In contrast, the CZI group, owing to a lack of DOX, relied solely on the chemodynamic activity of the CZ core, resulting in a relatively weaker signal. As shown in Figure 8b, under 808 nm laser irradiation at 1 W/cm^2^ for 10 min, the fluorescence intensity was significantly enhanced across most of the groups following the new order: CZID + Laser ≫ CZI + Laser > CZD + Laser > Control. The enhanced signal in the CZID + Laser group was attributed to a synergistic effect, where the ICG-derived photothermal effect accelerated the nanoplatform’s degradation, prompting a rapid release of both Cu^2+^ ions and DOX, as directly evidenced by the cumulative release kinetics profiles in Figure 6b. This coenhanced release behavior amplified ROS generation through combined photothermal, chemodynamic, and chemotherapeutic actions. While CZI and CZD groups lacked essential components for full synergy, they showed limited efficacy. Notably, the photothermal contribution from ICG in CZI was more responsive to laser irradiation than the purely chemotherapy-driven ROS induction from DOX in CZD, which would bring about a more pronounced reduction in the viability of MCF-7 tumor cells.

**Figure 8:**
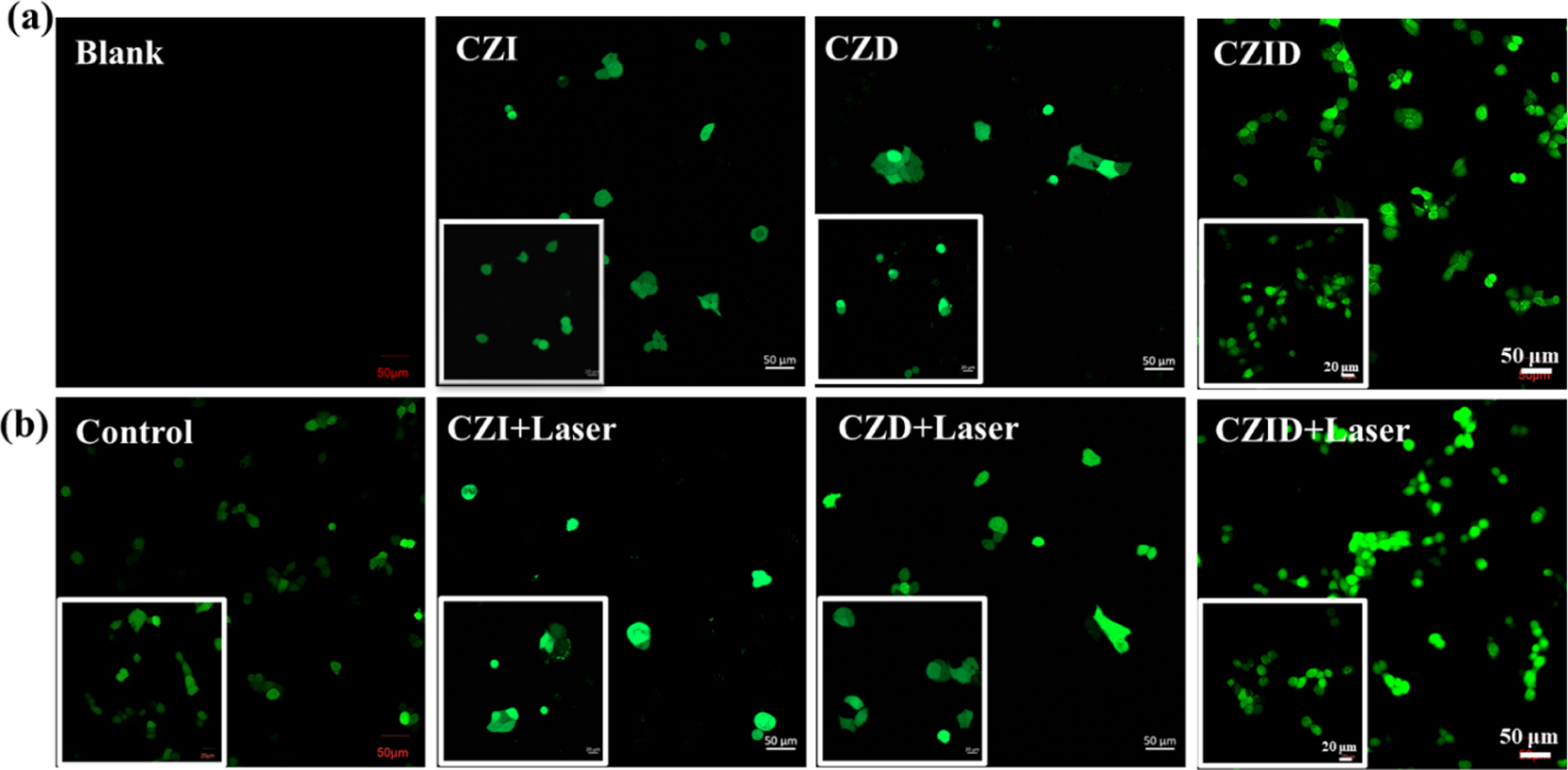
Representative CLSM images of intracellular ROS generation in MCF-7 cells as cocultured with CZI, CZD, or CZID (50 μg/mL): (a) without and (b) with 808 nm NIR irradiation (1 W/cm^2^, 10 min). Insets: 400× magnified view.

Figure 9 presents the live/dead fluorescence staining results of MCF-7 cells after coculturing with CZI, CZD, or CZID. It provided visual morphological evidence that the observed intracellular ROS elevation led to cell death. In the control, dense green fluorescence and intact cell morphology were observed within view, indicating a healthy cell state. After coculturing with different nanoplatforms, varying degrees of red fluorescence signals appeared across the groups. The CZID group exhibited a large number of red-fluorescent cells with obvious death characteristics, such as shrinkage and fragmentation, while the number of green viable cells decreased. The CZD group also showed significant red fluorescence, but its coverage and intensity were lower than those in the CZID group. In the CZI group, the red fluorescence signal was further reduced, appearing as scattered dots. The effectiveness in inducing cell death across the groups showed the following order: CZID > CZD > CZI. This trend was basically consistent with the ROS levels detected in Figure 8a, further indicating that the synergy between the specific components of CZID could achieve highly efficient killing of tumor cells.

**Figure 9:**
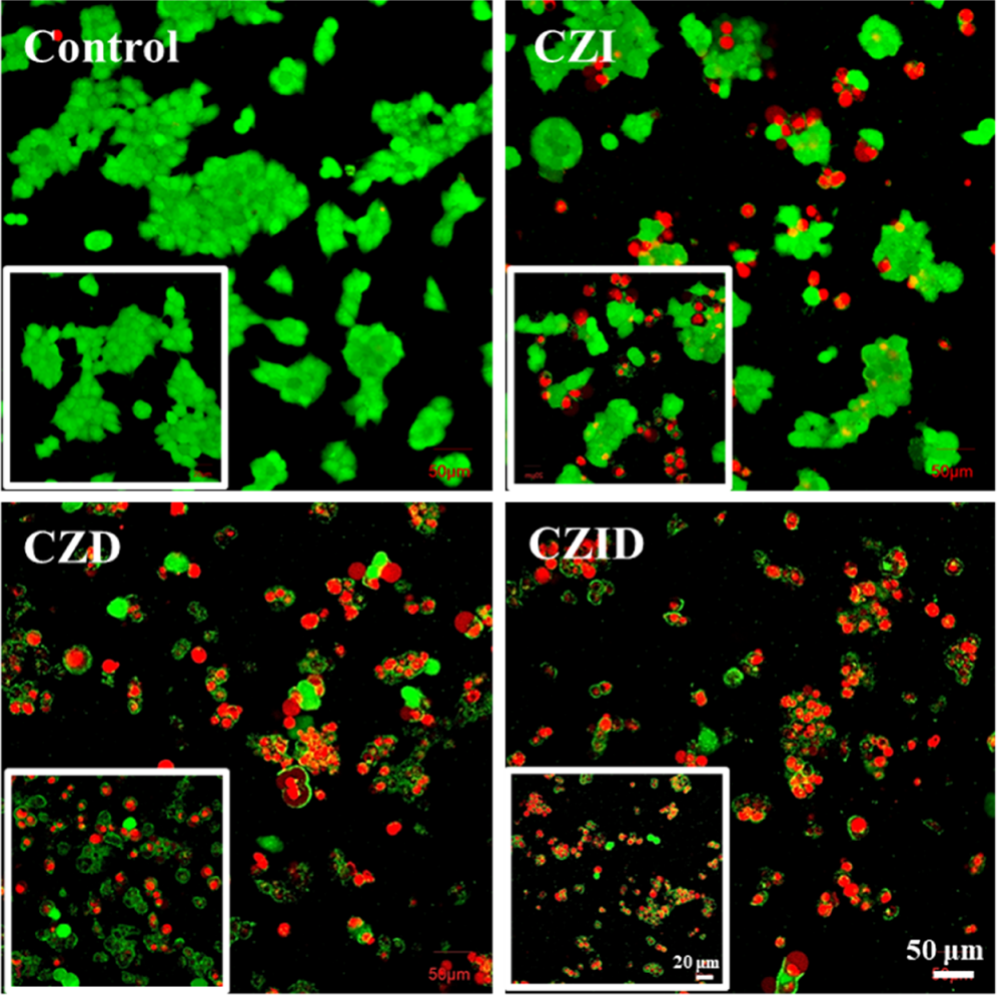
CLSM images of MCF-7 tumor cells stained with calcein-AM and PI after 24 h of coculturing with the CDI, CDZ, or CZID nanoplatform. Insets: 400× magnified view.

#### 3.3.3 Death/Survival Situations of Tumor Cells and Normal Cells

The lethal results on MCF-7 cells brought by the synthesized samples combined with supplementary operation were summarized in Figure 10a–c. As MCF-7 cells were cocultured with CZI, CZD, or CZID, the cell viabilities of all three nanoplatforms shown in Figure 10a were negatively correlated with the working concentration; they showed a dramatic decrease at 50 μg/mL and a minimum of less than 5% at 100 μg/mL. Moreover, the significant differences in cell viability among samples were only detected at the concentration of 50 μg/mL, where CZID caused more deaths, and its role in killing tumor cells can be attributed to two sources. One was the released Cu^2+^ ions from the structural collapse of CZ in the intracellular acidic environment; they experienced GSH reduction followed by Fenton-like oxidization to supply one ROS of ^•^OH free radicals, as supported by the explanations to Figures 5 and 8. The accumulated ROS could further destroy the cell membrane by predation of the negative charges on it and damage the mitochondria by denaturing proteins and inactivating enzymes,^39^ leading to irreversible injuries to tumor cells. The other was the released DOX drug, which, as a broad-spectrum antitumor antibiotic, acted not only on DNA but also on RNA.^40^ Its mechanism was figured out as the defunctionalization of DNA as a template for nucleic acid synthesis after embedding the double-helix base pairs, interfering with the transcription process and preventing the formation of mRNA,^41^ finally disrupting tumor cell regeneration and proliferation. In addition, DOX could further form semifree radicals in the presence of enzymes, which altered the permeability of cell membranes by binding to cell membrane phospholipids, performing antitumor functions similar to those of ^•^OH radicals.^42^ Accompanied by 10 min of laser irradiation, the viabilities of MCF-7 cells in Figure 10b showed trends consistent with those without laser irradiation, in terms of both sample and concentration. While limited by the low loading content, the temperature-rising role of ICG depended on the concentration of CZID, which only created a significant difference in cell viability at a suitable concentration, like 50 μg/mL; once below or above that, its effect was either not sufficiently pronounced or was overshadowed by the effects of CZ and DOX. Figure 10c further shows the differences among samples and additional irradiation on cell viability at the same concentration of 50 μg/mL. 10 min laser irradiation worked on all ICG-containing samples, resulting in a significant decrease in MCF-7 cells. According to the photothermal performance shown in Figure 4, NIR light exposure enables an FDA-approved photosensitizer to selectively heat the tumor microenvironment, with accumulated heat transferring into the cells to trigger cancer cell death by hyperthermia.^43^ Moreover, the photothermal effect of ICG could also change the permeability of the cancer cell membrane, which in turn improved the drug delivery from extracellular to intracellular, overall enhancing the chemotherapeutic efficacy of DOX.^44^ Thus, CZID had a stronger killing effect on MCF7 cells than CZI and CZD. Combining the results in Figures 5 and 8, the laser irradiation enabled the CZID nanoplatform to construct the synergistic relationships between the CZ material and the two drugs and between the ICG and the DOX in terms of accelerating the copper ions release and improving the DOX delivery under temperature increase, which resulted in a comprehensive enhancement of its antitumor ability.

**Figure 10:**
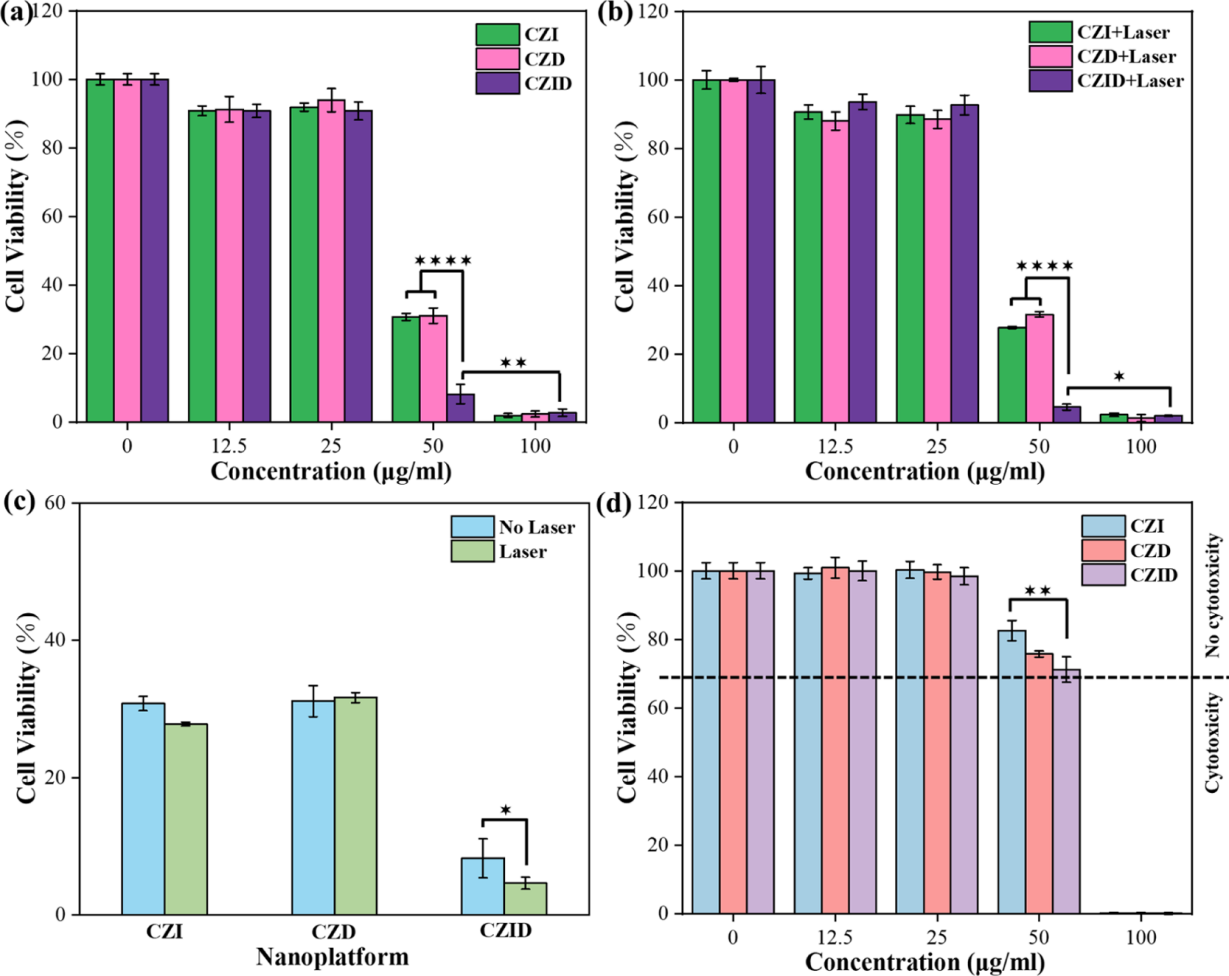
Cell viabilities after 24 h for those under various conditions: (a) MCF-7 cells cocultured with CZI, CZD, and CZID; (b) MCF-7 cells cocultured with target samples and irradiated by 808 nm laser (1 W/cm^2^); (c) same cells cocultured with 50 μg/mL of the same samples with or without laser irradiation; (d) MCF-10A cells cocultured with target samples. Error bars: SD.

Figure 10d depicts the viability of MCF-10A cells after 24 h of coculturing with the synthesized samples, which aimed to verify whether the killing actions on tumor cells threaten normal cell survival. It was found that the cell viability was close to 100% of the control at working concentrations of no more than 25 μg/mL, regardless of which sample. Although further increases in concentration resulted in significant decreases in cell viability, the values at 50 μg/mL were all beyond 70% with a reactivity of a slight level, which suggested that CZI, CZD, and CZID produced no cytotoxic effect on MCF-10A cells, as defined by ISO10993-2002. Unlike MCF-7 tumor cells, the neutral intracellular environment of MCF-10A cells rendered them insensitive to the chemodynamic process,^45^ and the suppressed GSH depletion further weakened the attack of the ^•^OH radical generated by the Fenton-like reaction on normal cells. Meanwhile, the metabolism of normal cells was not active enough, which allowed them to withstand the increased temperature of the cellular microenvironment to a greater extent, reducing the thermal damage derived from laser exposure.^46^ However, the lethal effects of free radicals and photothermia were merely diminished rather than absent, which still produced statistical differences on various nanosamples; the cell viabilities ranked in increasing order of CZI > CZD > CZID. Upon raising the concentration to 100 μg/mL, there was a corresponding increase in the release of Cu ions and loading of DOX, which led to an elevated number of free radicals that further compromised MCF-10A cells but were insufficient to cause massive death. The viability of MCF-10A cells was now less than that observed in MCF-7 tumor cells, indicating that other factors also contributed to the toxicity. Based on our previous studies on the cytotoxicity of nanometals and nano-oxides,^47^ one of the probable reasons was attributed to the excess release of metal ions. Compared to copper ions, zinc ions owned greater quantities since zinc itself was a major component of their ZIF-8 unit in all CZ-based samples. As a result of increasing concentration, the same increase in ionic amounts allowed for the excessive release of zinc at 100 μg/mL, replacing copper as the key factor fatal to normal cells.^48^

To further verify the broad-spectrum antitumor efficacy of the CZID nanoplatform, its cytotoxicity was also evaluated against 4T1 tumor cells. As depicted in Figure S3, CZID exhibited concentration-dependent killing effects, and these effects could be enhanced by 808 nm laser irradiation; this trend is consistent with the one observed in MCF-7 cells, supporting the broad applicability of CDIZ across tumor types. Comparative analysis further revealed that 4T1 cells exhibited higher sensitivity to the CZID coculturing combined with irradiation, showing statistically significant cell killing even at a lower concentration of 12.5 μg/mL. Such a difference in efficiency, rather than efficacy, is likely attributable to cell-specific characteristics such as differential cellular uptake rates or intrinsic metabolic activity.^49,50^

Generally, all components within the CZID system contributed to the establishment of a multimodal therapeutic nanoplatform that combines chemodynamic, photothermal, and chemotherapeutic modalities. The photothermal effect accelerated the degradation of ZIF-8, promoting the release of Cu^2+^ and DOX, which enhanced both chemodynamic and chemotherapeutic therapies, achieving a synergistic antitumor effect. Consequently, at 50 μg/mL, CZID induced intracellular ROS, hyperthermia, and DOX release, effectively eliminating MCF-7 cells while sparing MCF-10A normal cells. As systematically compared in Table 1, this favorable efficacy–safety profile at 50 μg/mL is highly competitive among recent nanoplatforms, with many lacking comparable normal cell data to define a complete therapeutic window. Although the CZID system has demonstrated promising anticancer potential and selective biocompatibility at the cellular level, its clinical translation requires further evaluation through systematic animal studies, with a focus on its in vivo behavior, pharmacokinetics, and long-term safety.

**Table 1:** Comparison of the In Vitro Performance between Our CZID Nanoplatform and Other Recently Reported Systems^a^ ^b^

		tested cell models			
nanoplatform	therapy type	tumor cells cytotoxicity	normal cells cytocompatibility	effective concentration	ref
MOF-Pt(IV)@GOx	CDT/chemo/ST	4T1, cell viability <10% at 25 μg/mL	not tested	not determined	^51^
Lip@Fe–Cu-MOFs	CDT/PTT	4T1, cell viability <30% at 50 μg/mL	mBM-Neu, cell viability >85% at 100 μg/mL	50 μg/mL	^52^
Fe_3_O_4_@SiO_2_@Cu	CDT/chemo	MCF-7, cell viability <20% at 50 μg/mL	BALB/3T3, cell viability >80% at 5 μg/mL	10–30 μg/mL	^53^
MH-PLGA-IR780	ferroptosis/PDT	HOS, cell viability <40% at 60 μg/mL under irradiation	not tested	not determined	^54^
Ag_2_S@CAT-Ce6@Oxa	PDT/PTT/chemo	HT29, cell viability <40% at 60 μg/mL under irradiation	not tested	not determined	^55^
MoS_2_-PB-TEG-FA	CDT/PTT	MCF-7, cell viability <55% at 50 μg/mL	HEK-293, cell viability >90% at 100 μg/mL	50 μg/mL	^56^
Cu-ZIF-8@ICG**&**DOX	CDT/PTT/chemo	MCF-7, cell viability <5% at 50 μg/mL under irradiation	MCF-10A, cell viability >70% at 50 μg/mL	50 μg/mL	our work

a Therapy type: chemo: chemotherapy; ST: starvation therapy; PDT: photodynamic therapy.

b Cell lines: mBM-Neu: mouse bone marrow-derived neutrophils; Balb/3T3: mouse embryonic fibroblasts; L929: mouse fibroblast cells; HFF: human foreskin fibroblasts; HOS: human osteosarcoma cells; HT29: human colorectal adenocarcinoma cells; HEK-293: human embryonic kidney cells.

## 4 Conclusions

For antitumor therapeutic applications, an “all-in-one” nanoplatform, Cu-ZIF-8 loaded with ICG&DOX (CZID), was successfully developed. Structural optimization revealed that 25% designed copper doping level yielded Cu-ZIF-8 nanoparticles with an ideal average size of 99.6 nm and an actual doping content of 7.46%. The dual loading of ICG and DOX induced a morphological transition to a spherical core–shell structure, achieving high loading capacities of 23.01 μg/mg and 122.43 μg/mg, respectively. The CZID nanoplatform exhibited excellent tumor microenvironment responsiveness, undergoing acid-triggered degradation to release active components. The released Cu^2+^ ions demonstrated their chemodynamic activity, depleting GSH and generating cytotoxic ^•^OH radicals. Under 808 nm laser irradiation, the loaded ICG enabled efficient photothermal conversion. Critically, the localized heat not only induced hyperthermia but also accelerated the release of Cu^2+^ ions and DOX, thereby synergistically enhancing both chemodynamic and chemotherapeutic efficacy. At an optimal concentration of 50 μg/mL, CZID induced intracellular ROS generation and triggered apoptosis in 95.36% of MCF-7 tumor cells while maintaining over 70% viability in MCF-10A normal cells. These in vitro results validate the CZID nanoplatform as a promising and effective strategy that achieves synergistic antitumor therapy by integrating chemodynamic, photothermal, and chemotherapeutic modalities.

## Supplementary Material



## Data Availability

All data supporting the findings of this study are available within the article and its Supporting Information.

## Notes

Detailed calculation method for the photothermal conversion efficiency (η), along with supplementary figures showing the visual comparison of as-synthesized nanoparticles, the UV–vis absorption spectra and standard curves for quantifying ICG and DOX, and the in vitro cytotoxicity of CZID against 4T1 cells in the presence and absence of laser irradiation (PDF)
