# Supplementary material for: Tumor Microenvironment-Responsive Nanoplatform of Cu-Doped ZIF‑8 Dual-Loaded with ICG and DOX for Photothermal-Enhanced Chemodynamic Therapy/Chemotherapy
Source: ACS Omega. 2026 Feb 11;11(7):12148–62. doi: 10.1021/acsomega.5c11491 (PMC12946994; doi:10.1021/acsomega.5c11491)
Supplement: Supplementary file 1 [file ao5c11491_si_001.pdf]

Supporting Information

Tumor microenvironment-responsive nanoplatform of Cu doped ZIF-8 dual-loaded with ICG and DOX for photothermal-enhanced chemodynamic therapy/chemotherapy

Tao Yang[a] Tao Wang[a] Tao Shen[a,b] Mingrong Dong[b] Jinkun Liu[a,*]

Ming Ni[c,] Yan Zhu[d,a ***]**

[a] Faculty of Materials Science and Technology, Kunming University of Science and Technology, Yunnan, 650093, China

[b] Faculty of Information Engineering and Automation, Kunming University of Science and Technology, Kunming 650500, China

[c] Department of Orthopaedics, Shanghai Key Laboratory for Prevention and Treatment of Bone and Joint Diseases, Shanghai Institute of Traumatology and Orthopaedics, Ruijin Hospital, Shanghai Jiao Tong University School of Medicine, Shanghai 200025, China.

[d] Siluzhisheng (Beijing) Intelligent Technology Co., Ltd., Miyun District, Beijing, 101500, China

[*] Corresponding authors:

E-mail addresses: bio-liujinkun@kust.edu.cn (Jinkun Liu),

gendianqing@163.com (Ming Ni), and zhuyan@kust.edu.cn (Yan Zhu).

Section S1. Calculation of Photothermal Conversion Efficiency

The section provided the complete calculation method for the photothermal conversion efficiency (***η***) of the CZID nanoplatform, as mentioned in Section 2.5 of the main text. The calculation followed the energy balance method established by [21]. The calculation formulas were displayed below:

$$\eta = \frac{hS\Delta T_{max,\,sample} - hS\Delta T_{max,\,water}}{I \times \left(1 - 10^{-A}\right)} \tag{1}$$

$$hS = \frac{C \times m}{\tau_s}, hS_{water} = \frac{C \times m_{water}}{\tau_{s,water}} \tag{2}$$

$$hS = \frac{C \times m}{\tau_s}, hS_{water} = \frac{C \times m_{water}}{\tau_{s,water}} \tag{3}$$

$$\theta = \frac{T - T_{surr}}{\Delta T_{max}}, \theta_{water} = \frac{T_{water} - T_{surr}}{\Delta T_{max,water}} \tag{4}$$

Where η represented the photothermal conversion efficiency of the CZID nanocomposite, ***h*** was the heat transfer coefficient, ***S*** was the surface area of the container, and ΔT_{max} and $\Delta T_{max,\,water}$ were the maximum temperatures of the CZID suspension and water under 808 nm laser irradiation for 10 min, respectively. ***I*** showed the power density of the 808 nm laser irradiation, ***A*** meant the absorbance of the CZID suspension at 808 nm, ***C*** was the heat capacity of the suspension, ***m*** and m_{water} were the masses of the CZID suspension and water, respectively, and τ_s and $\tau_{s,\,water}$ were defined as the slope obtained by fitting the cooling curve of the CZID suspension and water, respectively. ***T*** and T_{water} were the temperature variations of the CZID suspension and water recorded at different irradiation times. T_{surr} was considered as the ambient temperature.

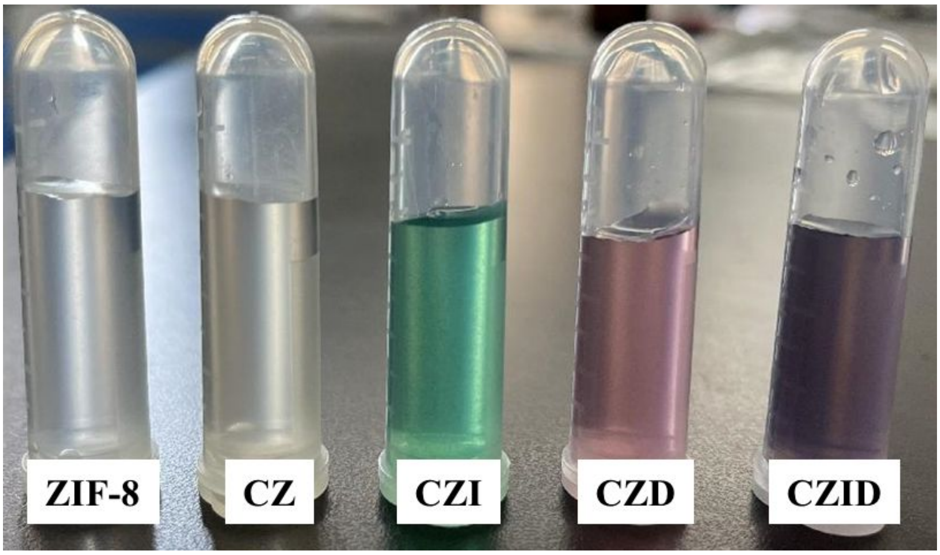

Figure S1. Photographs of ZIF-8, CZ, CZI, CZD, and CZID after redispersion in deionized water for color change comparison

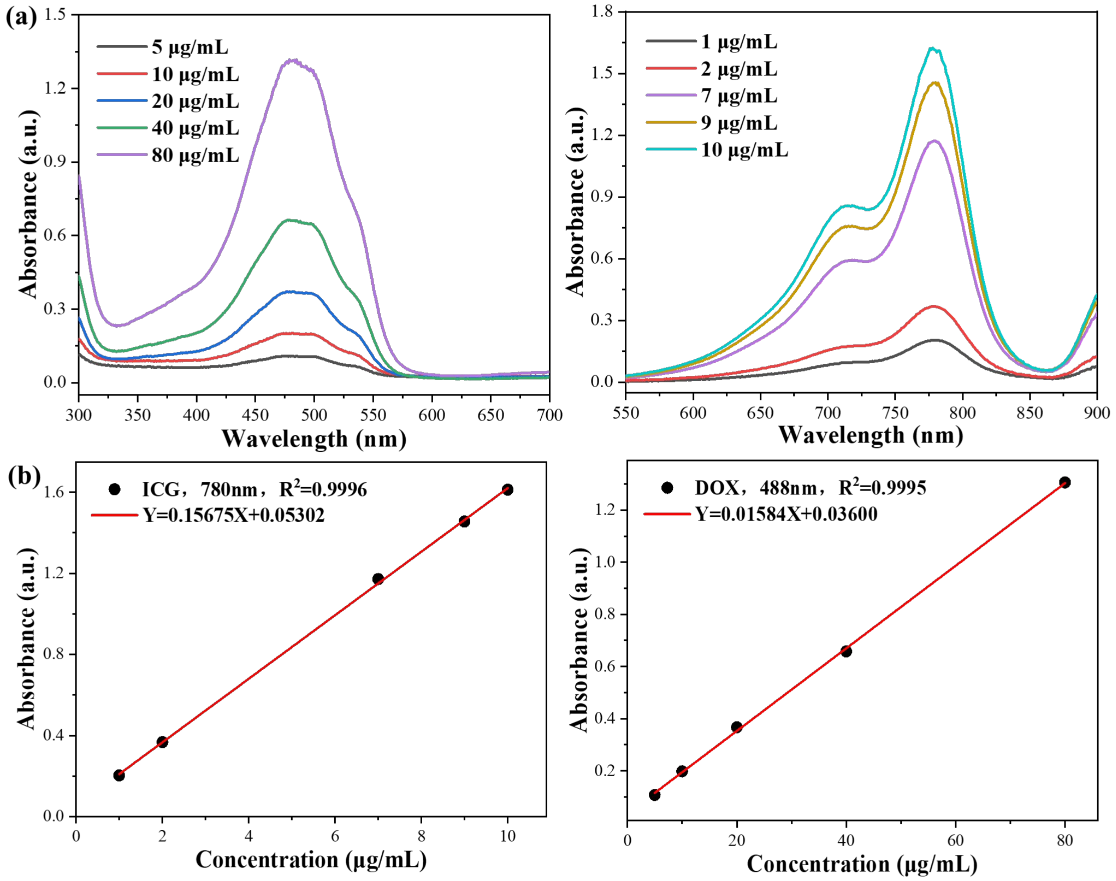

Figure S2. (a) UV-vis absorption spectra of ICG (left) and DOX (right) dissolved in deionized water under nominal concentrations; (b) the fitted absorbance-concentration standard curves and resulting linear equations of the two drugs

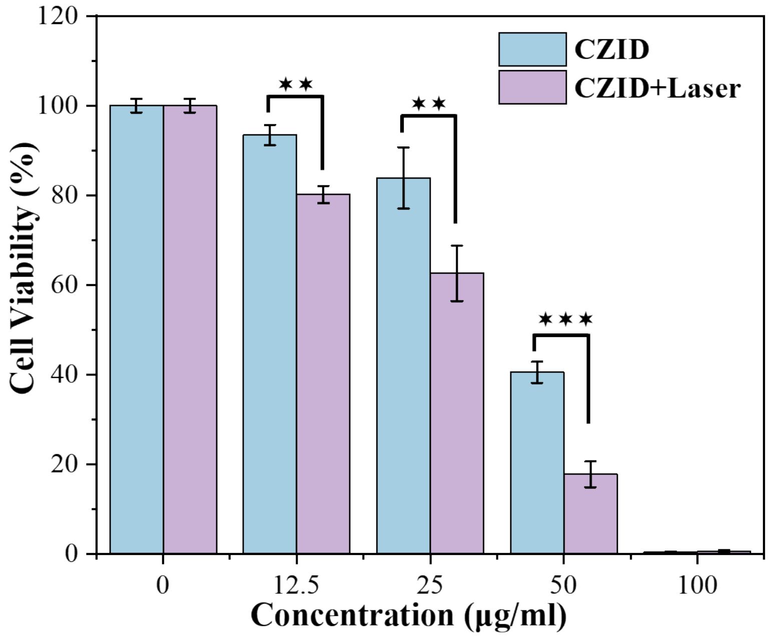

Figure S3 Cell viability of 4T1 tumor cells as co-cultured with CZID at the designed concentrations and 808 nm NIR irradiation, error bars: SD
